# Collocation of avian and mammal antibodies to develop a rapid and sensitive diagnostic tool for Russell's Vipers Snakebite

**DOI:** 10.1371/journal.pntd.0008701

**Published:** 2020-09-21

**Authors:** Jing-Hua Lin, Che-Min Lo, Ssu-Han Chuang, Chao-Hung Chiang, Sheng-Der Wang, Tsung-Yi Lin, Jiunn-Wang Liao, Dong-Zong Hung

**Affiliations:** 1 Graduate Institute of Veterinary Pathobiology, National Chung Hsing University, Taichung, Taiwan; 2 Division of Toxicology, China Medical University Hospital, Taichung, Taiwan; 3 Changhua Animal Propagation Station, Livestock Research Institute, Council of Agriculture, Executive Yuan, Changhua, Taiwan; Institut de Recherche pour le Développement, BENIN

## Abstract

Russell’s vipers (RVs) envenoming is an important public health issue in South-East Asia. Disseminated intravascular coagulopathy, systemic bleeding, hemolysis, and acute renal injury are obvious problems that develop in most cases, and neuromuscular junction blocks are an additional problem caused by western RV snakebite. The complex presentations usually are an obstacle to early diagnosis and antivenom administration. Here, we tried to produce highly specific antibodies in goose yolks for use in a paper-based microfluidic diagnostic kit, immunochromatographic test of viper (ICT-Viper), to distinguish RVs from other vipers and even cobra snakebite in Asia. We used indirect ELISA to monitor specific goose IgY production and western blotting to illustrate the interaction of avian or mammal antibody with venom proteins. The ICT-Viper was tested not only in prepared samples but also in stored patient serum to demonstrate its preliminary efficacy. The results revealed that specific anti-*Daboia russelii* IgY could be raised in goose eggs effectively without inducing adverse effects. When it was collocated with horse anti-*Daboia siamensis* antibody, which broadly reacted with most of the venom proteins of both types of Russell’s viper, the false cross-reactivity was reduced, and the test showed good performance. The limit of detection was reduced to 10 ng/ml *in vitro*, and the test showed good detection ability in clinical snake envenoming case samples. The ICT-Viper performed well and could be combined with a cobra venom detection kit (ICT-Cobra) to create a multiple detection strip (ICT-VC), which broadens its applications while maintaining its detection ability for snake envenomation identification. Nonetheless, the use of the ICT-Viper in the South-East Asia region is pending additional laboratory and field investigations and regional collaboration. We believe that the development of this practical diagnostic tool marks the beginning of positive efforts to face the global snakebite issue.

## Author summary

For a long time, many scientists have made efforts to develop more effective antivenoms or to expand the limits of existing antivenoms. Such endeavors are challenging but worth the effort in the long run. However, to use precious antivenom more effectively, a snake species discrimination tool might be helpful for treatment by ensuring accurate antivenom usage and dosage. In this study, we revealed that avian IgY might be an economical alternative for collocation with the equine F(ab’)_2_ fragment in the development of a diagnostic device with improved performance. Only a few avian eggs (about 10–15) would be necessary to produce 10,000 kits. Immunochromatographic test (ICT) is a simple, easy, fast, and low-cost point-of-care lateral flow assay device, and especially adapted to perform in resource-deficient areas. Such an antibody collocation strategy might be beneficial to overcome the urgent need for snakebite diagnosis as soon as possible. More cooperative investigations across Asia continent might be initiated in the future to face the challenge of snakebites together.

## Introduction

The snakebite of Russell’s vipers (RVs), *Daboia (D*.*) russelii* (western RV, RVW) and *D*. *siamensis* (eastern RV, RVE), is an important public health issue in South-East Asia due to its wide but discrete distribution and notable organs injuries, such as neurological paralysis, bleeding diathesis and acute kidney injury in cases of significant envenomation. RVs were previously recognized as one species, but this has now been revised to two species according to the biogeographical distribution of the eastern and western clades, which are divided by a mountain ridge located in northwest Burma [1, 2]. There are few differences in the envenomation presentations of the two species. Patients present with local swelling, consumptive coagulopathy with severe bleeding, hypopituitarism, and acute renal failure when bitten by the RVE, while those bitten by the RVW also present with neurological paralysis [3, 4]. Each key clinical manifestation needs to be differentiated from those of other offending snakes due to the abundance and biodiversity of venomous snakes in these areas [5]. The same as Taiwan, RVE envenomation presented with local swelling, systemic coagulopathy and neurotoxicity, and should be differentiated from envenomation of *Naja atra* (NA), *Protobothrops mucrosquamatus* (PM) and *Trimeresurus stejnegeri* (TS) [6–8]. It may be easy to obtain good results if we can identify the offending snake by examining the killed specimen, correctly identifying figures or finding venom in the wound or in patient biosamples according to the diagnostic flow-chart of snakebite management guidelines published by the World Health Organization (WHO). The syndromic approach has been applied in different ways on different continents and is thought to be useful sometimes; nonetheless, delayed diagnosis and late or inadequate antivenom therapy are frequently encountered and are a concern [9, 10]. A simple, sensitive and high efficacy diagnostic method might be needed to reduce the harm caused by misdiagnosis, especially in regions with poor health systems or limited medical resources.

Snake venom is a mixture of toxic and nontoxic proteins that cause a variety of complex symptoms [11]. Since the 19th century, specific antivenom raised against venomous snakes has been considered the most effective therapy for venomous snake bites. Previous studies have shown that early antivenom treatment of snakebites might reduce the severity of thromboses and systemic bleeding, the incidence of acute renal failure, and the length of the recovery time [12–14]. These observations are consistent with our clinical experiences; furthermore, the use of antivenom within 3–6 hours did result in better clinical outcomes for RVE snake bites in Taiwan [8]. Early treatment is closely related to early diagnosis. The cardinal hemotoxic signs of RVs snake bite, such as thrombocytopenia and coagulopathy, could be evaluated by the 20 Minute Whole Blood Clotting Test (20WBCT) [15, 16]. Failure of the blood to clot in a clean glass tube after 20 minutes has been considered evidence of severe hypofibrinogenemia and rules out elapid snake bite in the Asia region [5–7, 17, 18]. However, consumptive coagulopathy might occur after most Viperidae snake bites in this region, and neurotoxic manifestations in cases of RVW envenoming could be mistaken for symptoms caused by cobra or other Elapidae snake bites. All of these findings highlight that accurate differentiation is urgently needed to provide adequate doses of selective antivenom and prevent further complications from RVs snake bites [5, 10].

The major components of snake venoms are proteins or peptides that induce specific toxicities in other animals and humans as well as immunologic reactions. Theakston et al. first reported the use of enzyme immunoassays to detect snake venom and venom antibodies in the sera of experimental animals and human victims [19]. We also developed ELISA methods to confirm and detect cobra or RVE snake bites in Taiwan [8, 20]. ELISA has several disadvantages, especially the need for time-consuming procedures. Previously, we developed a rapid diagnosis kit according to the principle of immunochromatography to study cobra bites [21]. The kit used a lateral flow assay (LFA) with an immunochromato-microfluidic paper-based device that has been widely used to detect pregnancy, drugs, bacteria, viruses, and cardiac biomarker as the technique has advanced [22–27]. Based on this LFA method, we could differentiate cobra snake bites from other viper bites quickly in the same area [21]. Recently, exploiting of avian egg yolk antibodies (IgY) showed several advantages over that of conventional mammalian antibody production [28]. Avian IgY has taken the advantages of low costs, high yield, and long-term stability at 4°C when applied for LFA [29, 30]. A few studies have used avian IgY in immunochromatography to assay simple proteins, but IgY has not been applied in the development of complex venom detection [31–34]. Furthermore, owing to phylogenetic distance, IgY does not react with human rheumatoid factors, which are the major cause of interference that leads to false positives in many immunoassays [35]. We believe that the IgY antibodies used in the immunochromatography strip might improve its sensitivity and specificity.

To date, the WHO has paid more attention to the public health problems that result from venomous snakebites and recognized once again that such problems represent a neglected tropical disease. The 71st World Health Assembly adopted the WHA71.5 resolution on snakebite envenoming, which gave the WHO a powerful mandate to address this neglected tropical issue. To contribute to our efforts to help achieve the global objective of halving the incidence of death and disability due to snakebite before 2030 [36], the aim of this study was to raise specific goose IgY against RVW venom protein and collocated it with equine anti-RVE F(ab’)_2_ in the ICT-Viper to meet the unmet medical need for the differential diagnosis of RVE bite to distinguish it from envenomation caused by other venomous snakes. We demonstrated the limit of detection and good accuracy of ICT-Viper and tested it with some stored human samples to verify the possibility for further larger-scale clinical trials. Furthermore, we also tested the feasibility of multi-detection by using a single strip, which might allow broad-spectrum venom identification with a single membrane in the future.

## Materials and methods

### Ethics statement

All animals were kept in individual cages with access to water and food ad libitum at the Changhua Animal Propagation Station, Livestock Research Institute, Council of Agriculture. According to animal well-being guidelines, the geese were strictly maintained under the appropriate conditions. The procedure (protocol No. 2016–420) was reviewed and approved by the Institutional Animal Care and Use Committee (IACUC) of China Medical University, Taichung, Taiwan.

Human serum samples were collected after the patient signed the informed consent form. The use of human specimens was carried out after the protocol (protocol No. CMUH107-REC1-005) was reviewed and approved by the Research Ethics Committee (REC) I of China Medical University Hospital, Taichung, Taiwan.

### Materials and chemicals

All chemicals used in this study were of analytical grade and purchased from Sigma-Aldrich (St. Louis, MO, USA), including the lyophilized RVW snake venom (product ID: V2501). The venom of *Naja atra* (NA) was purchased from Latoxan (Portes-lès-Valence, France). Other snake venoms, such as those from *Protobothrops mucrosquamatus* (PM) and *Trimeresurus stejnegeri* (TS), as well as the equine antivenom of RVE (>1000 Tanaka unit) were all donated by the Centers for Disease Control (CDC), Taiwan. Rabbit and mouse polyclonal antibodies against goose IgY were purchased from Abcam (Cambridge, GBR) and MyBioSource Inc. (San Diego, USA), respectively. Peroxidase-conjugated goat anti-rabbit IgG (H+L), goat anti-mouse IgG (H+L), and goat anti-horse IgG (H+L) were purchased from Jackson ImmunoResearch Inc. (West Grove, PA, USA). The material used for the antigen-immobilized CNBr-activated Sepharose 4 Fast Flow was purchased from GE Healthcare (Chicago, IL, USA). The Vivaspin 15 Turbo Centrifugal Concentrator was obtained from Sartorius (Göttingen, GER). Bolt 4–12% Bis-Tris Plus Gels and SeeBlue Plus2 Prestained Protein Standard were purchased from Thermo Fisher Scientific Inc. (Waltham, MA, USA). The Immobilon-PSQ PVDF membrane and the chemiluminescent HRP substrate used in western blotting were purchased from Merck Millipore (Burlington, MA, USA). Flat-bottomed microtitration plates were purchased from Corning Inc. (Corning, NY, USA). The nitrocellulose membrane (AE99) and the absorbent pad (grade 205) used for strip assembly were purchased from Whatman plc (Kent, GBR). Other components, such as the conjugate pad (grade 6613) and sample pad (grade 8964), were purchased from Ahlstrom-Munksjö (Helsinki, FIN).

### Goose immunization

To minimize the adverse effects of immunization and to produce specific antibodies efficaciously, we used a modified protocol origin from mammalian immunization with a strategy of low dose, low volume, and multisite induction [37, 38]. Venom was detoxified by adding and mixing one-tenth volume of 2.5% glutaraldehyde solution to final concentration of 0.25% at room temperature and incubated for 1 h before use.

**Fig 1:**
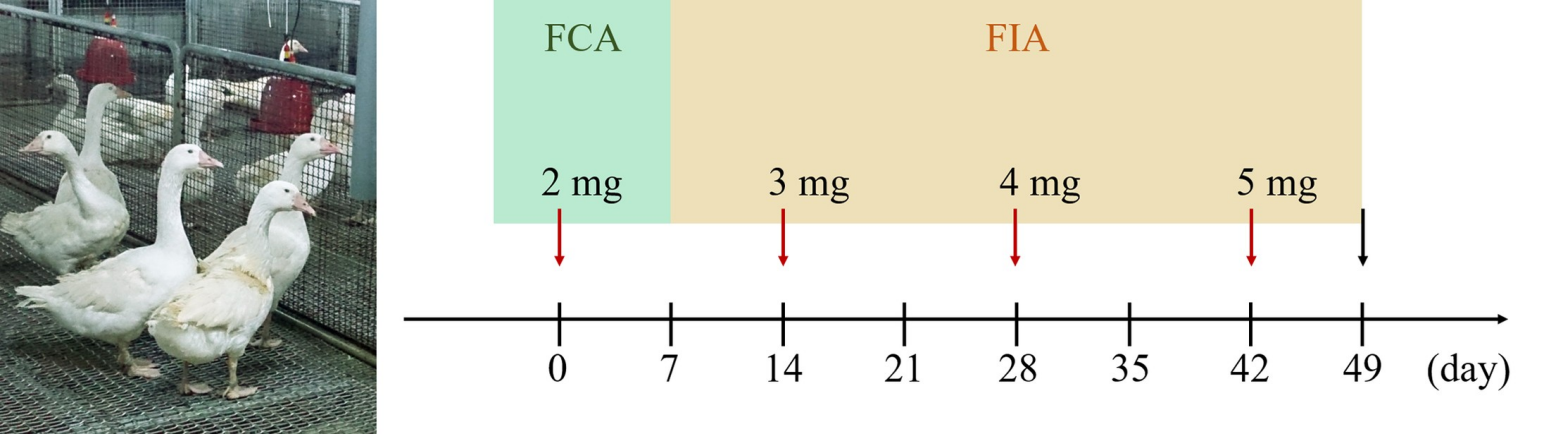
The time frame of the goose antibody induction process. Before the termination of immunization (black arrow), a total of four inoculations were performed within 49 days, once every 14 days (red arrow). For each inoculation, the detoxified venom was emulsified with either Freund’s complete adjuvant (FCA) or Freund’s incomplete adjuvant (FIA). The image of geese was obtained at the Changhua Animal Propagation Station.

The minimal disease (MD) grade of white Roman goose (4.5–5.0 kg, n = 3) was inoculated intramuscularly (i.m.) at four sites in the pectoral muscle (0.5 ml/site) with detoxified RVW venom (2 mg, day 0), which was emulsified with an equal volume of Freund’s complete/incomplete adjuvant (FCA/FIA). The booster injections were administered on Days 14, 28 and 42 at increasing dosages (3–5 mg) (Fig 1). To assay the immune response, goose blood was drawn from the brachial wing vein before the first inoculation and every 7 days during the immunization process. The laid eggs were collected every morning by feeding staff. The immunization was terminated when the serum titer had increased to a stable level (day 49) (Fig 1). Under humane anesthesia, all geese were sacrificed, and blood was collected by cardiac puncture. The blood samples were centrifuged (2,500 rpm for 5 minutes at 4°C) and stored at -20°C in serum form. All eggs were carefully and properly stored at 4°C until use.

### Antibody purification with an antigen-affinity system

The yolks of the eggs were pooled together, and the hydrophobic components (e.g., lipids and lipoproteins) were subsequently removed by a method of water dilution and salt precipitation described by Wallmann et al., with modifications [39]. First, the yolk was separated from the egg white, diluted and mixed with 20 volumes of distilled water, and the pH was adjusted to 5 by using 1 N HCl. For delipidation, the solution was subjected to a freeze-thaw cycle and centrifuged (16,000 g, 25 minutes at 4°C) to remove the hydrophobic precipitate. The goose polyclonal antibody was drawn out from the supernatant after ammonium sulfate precipitation and repeatedly passed through an antigen immobilization column generated with 5 mg of RVW venom on a CNBr-activated Sepharose 4 Fast Flow column (GE Healthcare, USA). After the nonspecific antibodies were washed out with 35 ml phosphate washing buffer, the venom-binding goose IgY was harvested with elution buffer (0.1 M glycine-HCl buffer, pH 2.3) and neutralized with 0.2 M pH 8.0 sodium phosphate buffer. The neutralized eluent was further dialyzed and concentrated with a Vivaspin 15 Turbo Centrifugal Concentrator (Sartorius, GER) to generate the conjugation eluent, which was used for subsequent tests and applications. The horse anti-RVE F(ab’)_2_ used for colloidal gold conjugation for the ICT-Viper was also purified with the same procedure.

### Indirect enzyme-linked immunosorbent assay (Indirect-ELISA)

A 96-well polystyrene microplate (Corning, USA) was coated with 0.4 μg/ml venom, which was dissolved in the coating buffer (50 mM carbonate/bicarbonate buffer, pH 9.6) at 4°C overnight. The next day, the plate was washed five times with 150 μl PBST (0.01% Tween-20 in PBS), and 100 μl blocking buffer (PBST containing 1% BSA) was added to each well, after which the plate was incubated for 1 h at 37°C. After washing, the samples of interest (e.g., goose serum or purified antibody) were subjected to serial dilution (from 1:500–1:256000; 1:500 was equivalent to 10 μg/ml) in PBST buffer, after which they were added to the plate and incubated. Subsequently, the plate was washed again and loaded with secondary antibody for 1h incubation, then followed by adding HRP-conjugated antibody prior to another incubation. After the last wash with PBST to remove unbound antibodies, the chromogenic reaction was performed with peroxidase substrate (0.4 mg/ml *o*-phenylenediamine dihydrochloride, 0.4 mg/ml urea hydrogen peroxide, and 0.05 M phosphate-citrate, pH 5.0) in the dark and terminated by the addition of 2 N sulfuric acid. The absorbance was measured at 492 nm using a Multiskan FC Microplate Photometer (Thermo Fisher Scientific, USA). If the absorbance ratio of the sample/blank exceeded 2.1, the highest sample dilution factor was defined as the ELISA titer; for example, the sample/blank ratio was 2.7 (which was higher than 2.1) when the highest dilution was 1:32,000, so the ELISA titer was defined as 32,000. All data were repeated for at least three times and shown as mean ± SD. The statistical analyses were performed using student’s t-test to calculate the *p*-values. Results with *p* < 0.05 were considered to be significant.

### Sodium dodecyl sulfate-polyacrylamide gel electrophoresis (SDS-PAGE) and western blotting

The protein compositions of snake venom (5 μg) were evaluated by a Bolt 4–12% Bis-Tris Plus Gel electrophoresis system (Thermo Fisher Scientific, USA). The samples were prepared with sample buffer in nonreduced conditions according to the manufacturer’s instructions. Novex Sharp Prestained Protein Standard (3.5–260 kDa) was used as a protein molecular weight marker and loaded in every gel at the same volume as that of the samples. The gel was run at a constant voltage (75 V) for 2 hours to separate each protein band well. After electrophoresis, the gels were soaked and stained in 0.1% Coomassie Brilliant Blue solution containing 50% methanol and 10% acetic acid and shaken for 1 h at room temperature. Afterward, the solution was replaced with the destaining solution (40% methanol and 10% acetic acid) to remove the residual dye until the gel background was nearly clear.

For western blotting, at the end of electrophoresis, the gels were placed in the electroblotting apparatus adjacent to a 0.22 μm PVDF membrane in buffer to transfer the protein from the gel to the membrane. Then, the membranes were soaked sequentially in the following solutions: blocking buffer (5% skimmed milk in PBST), primary antibody solution (i.e., horse anti-RVE F(ab’)_2_ in PBST, or goose anti-RVW IgY and secondary antibody as followed in PBST), and HRP-conjugated secondary antibody solution for 1 h. The proteins recognized by the horse or goose immunoglobulin were probed by using a chemiluminescent HRP substrate and detected by an ImageQuant LAS 4000 system (GE Healthcare, USA).

### Conjugation of colloidal gold and antibody

The affinity-purified horse polyclonal antibody (1 mg) was adjusted to approximately pH 8.2 with 0.1 M potassium carbonate and subsequently mixed gently with gold nanoparticles (30 nm diameter, OD1) solution (Sigma-Aldrich Co Ltd, USA) at room temperature for 45 minutes. After complete mixing, the gold-antibody (Au-Ab) conjugation was blocked by the addition of BSA solution at a final concentration of 2.5% and centrifuged for 20 minutes at 12,500 rpm and 4°C. The supernatant was discarded, and 1 ml of Tris-base buffer was added to wash the pellets several times. Finally, the final precipitates were carefully suspended in 600 μl Tris-BSA buffer and stored at 4°C.

### Preparation of the immunochromatographic test strip (ICT-Viper)

**Fig 2:**
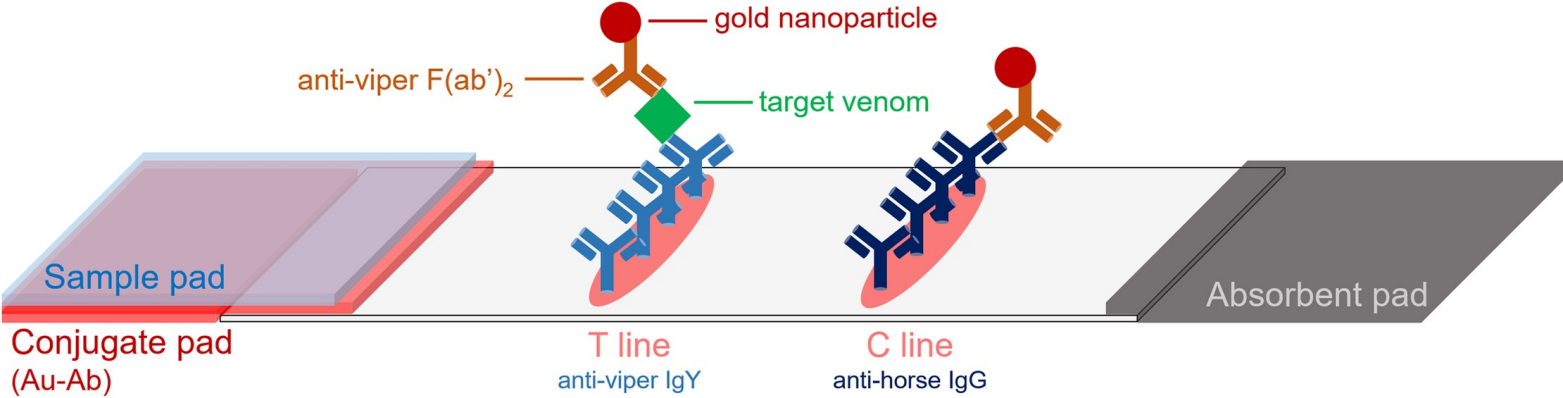
Schematic diagram of ICT-Viper for RVs venom identification. C line: control line, which is used for checking the immunochromatographic function; T line: test line, which is used for detecting the presence of venom in the sample. Au-Ab: gold-antibody conjugated particle, which is dispensed in the conjugate pad.

The immunochromatographic test strip used in this study was modified and assembled based on our previous study [21]. The conjugate pad and sample pad were soaked in 0.1 M borate buffer (pH 8.2) with 1% BSA and then dried at 37°C overnight. The prepared Au-Ab solution was added onto the conjugate pad, which was further dried in an oven for 40 minutes. Before assembling all the components, every pad was cut into 0.5 mm-wide pieces, which were placed on either side of the nitrocellulose membrane and pasted to the support card. The test line on the nitrocellulose membrane was loaded with 2.5 μg (2.5 mg/ml) goose anti-RVW IgY, and the control line was loaded with 0.8 μg (0.8 mg/ml) goat anti-horse IgG (Fig 2). The strips were freshly prepared before use to ensure immunochromatographic function.

### Performance of ICT-Viper *in vitro* and clinical cases

For checking the performance of ICT-Viper, all samples were tested with standard procedure as follows. Venom samples were freshly dissolved in fetal bovine serum (FBS) at concentrations of 0, 5, 10, 50, and 500 ng/ml to mimic the viscosity of human serum. Healthy human serum was used as the control while assaying the clinical samples. Total 90 μl of venom or clinical sample was loaded onto the sample pad of ICT-Viper. During the entire testing process, the strip must stand on a flat table, and avoid any shaking. The result was interpreted by the naked eye after 25 minutes and took photo immediately.

## Results

### The induction of goose polyclonal antibody production without significant adverse impacts

**Fig 3:**
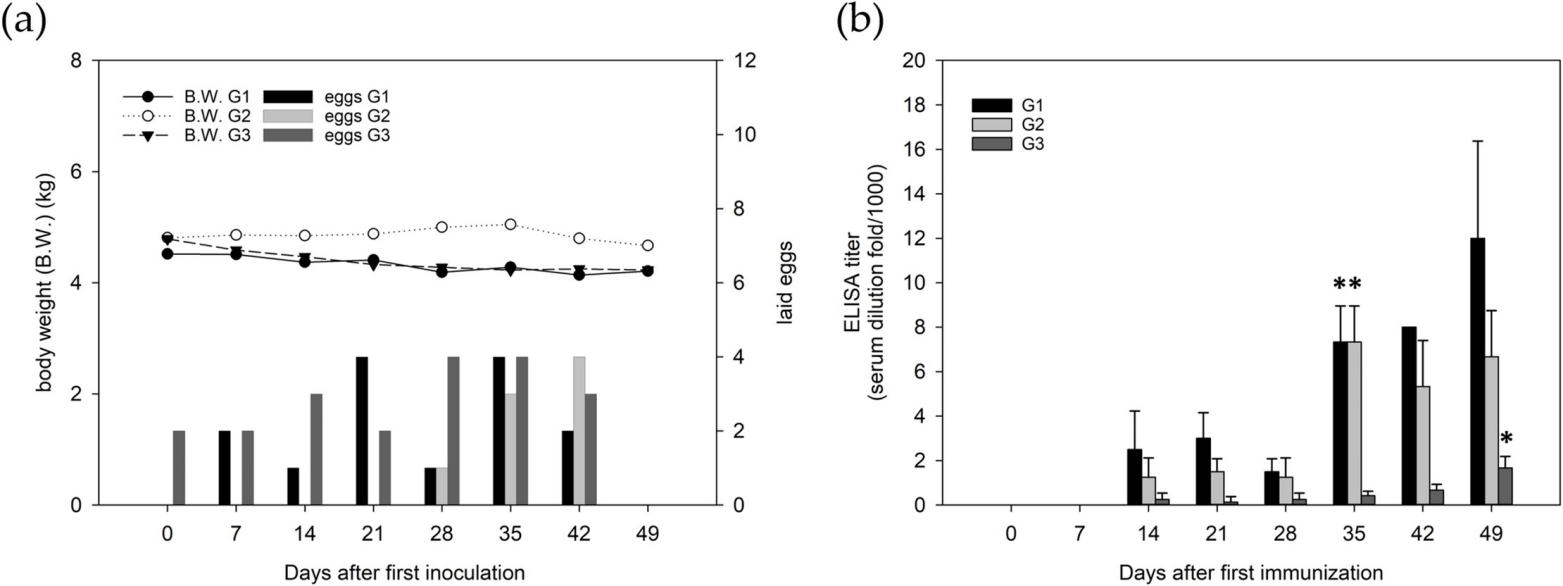
(a) Body weight and egg laying every week during the course of immunization. (b) ELISA titers of geese serum as the indication of the immune response for producing antivenom on various days. Results were the average of at least three repeats with standard deviation. The asterisk (*) represents an ELISA titer with a significant (*p* < 0.005) increase compared to that on Day 14. G1-3 indicates the number of the three geese used in the study. The geese were sacrificed on day 49.

During the course of immunization, the geese did not present any obvious changes in their appetite or body weight. The conditions of egg laying were also unaffected after the first inoculation (day 0) and those on successive days (days 14, 28 and 42) with increased dosages (Fig 3A). As shown in Fig 3B, we found that the antibody titer against RVW venom in geese serum was detected and increased starting on the 14th day, and it increased significantly on the 35th day, except for the titer in goose G3. Goose G3 showed a low immune response until day 49, which might have resulted from individual differences. Under the consideration of economic benefit and efficacy maximization, we chose the 20 eggs laid on days 35–49 for further analysis and application.

### Yolk antibody extraction by antigen-affinity purification

**Fig 4:**
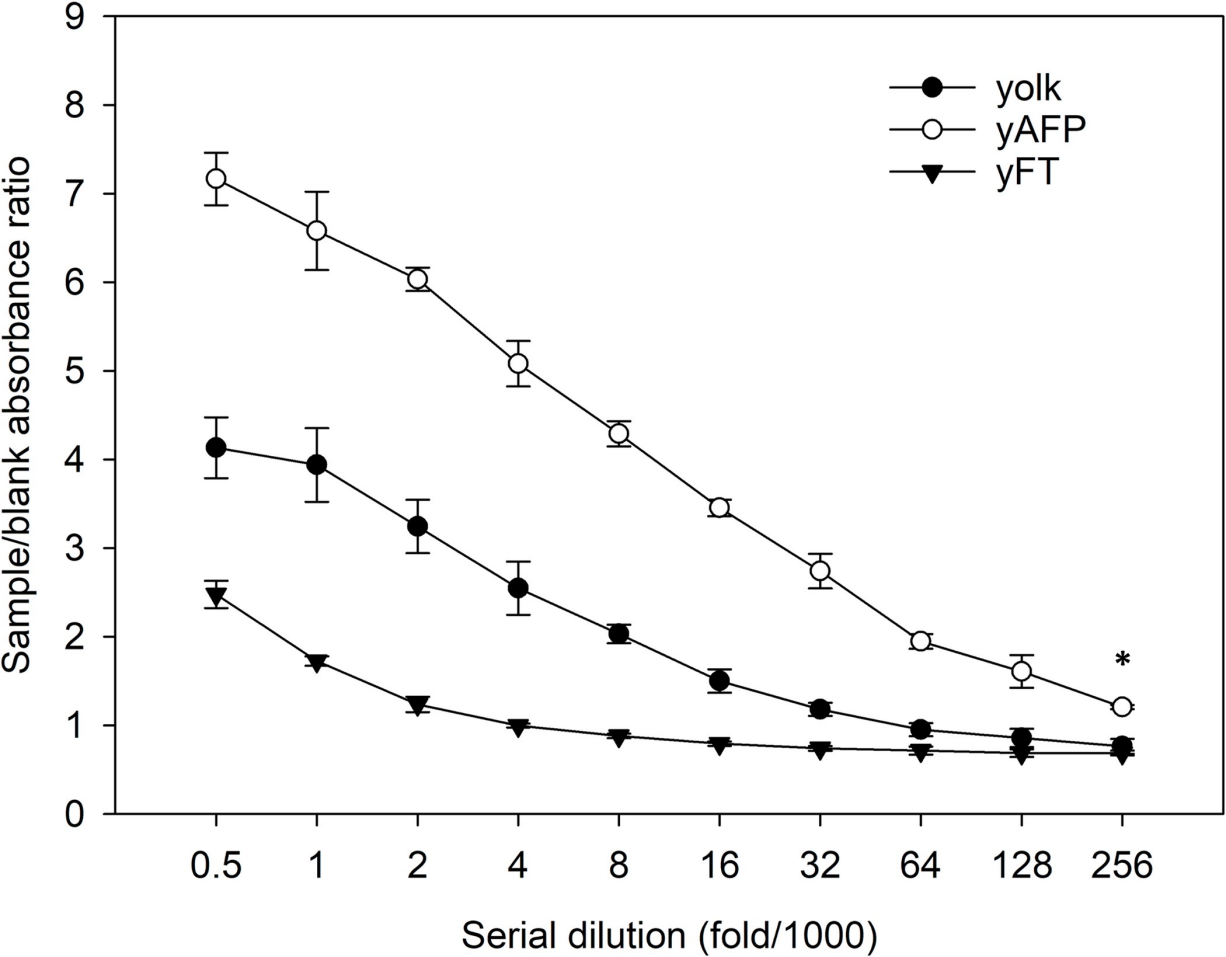
The antibody profiles of the delipidated yolk (yolk) as well as the flow-through solution (yFT) and eluted solution (yAFP) after antigen-affinity purification, which were determined by indirect ELISA. The eluted solution contained more specific goose anti-*D*. *russelii* (RVW) IgY per unit of protein than the other prepared samples, even at the highest sample dilution (1:256,000) (*, *p* < 0.005). The data are represented as the mean ± SD.

To obtain better performance in the diagnostic device, we purified the yolk antibody by antigen-specific affinity purification, and the results are shown in Fig 4. The degree of purification was tested by indirect ELISA in three kinds of samples from the solutions obtained before and after passage through the antigen-immobilized column and is represented as the sample/blank absorbance ratio versus the fold dilution. We found that the eluted solution (yAFP) had a higher quantity of antibodies specific to RVW venom than the flow-through solution (yFT) and the unpurified but delipidated sample (yolk). Even at the highest dilution (1:256,000), there were still more specific antibodies per microgram of protein (*p* < 0.005) in the yAFP than the other solutions. The purified goose anti-RVW IgY was applied in the following LFA development experiment to prove our purification protocol is suitable for goose IgY extraction. The amount of purified goose IgY was calculated to be 2.77 mg per egg in average.

### Goose and horse polyclonal antibodies react with venom proteins differently

**Fig 5:**
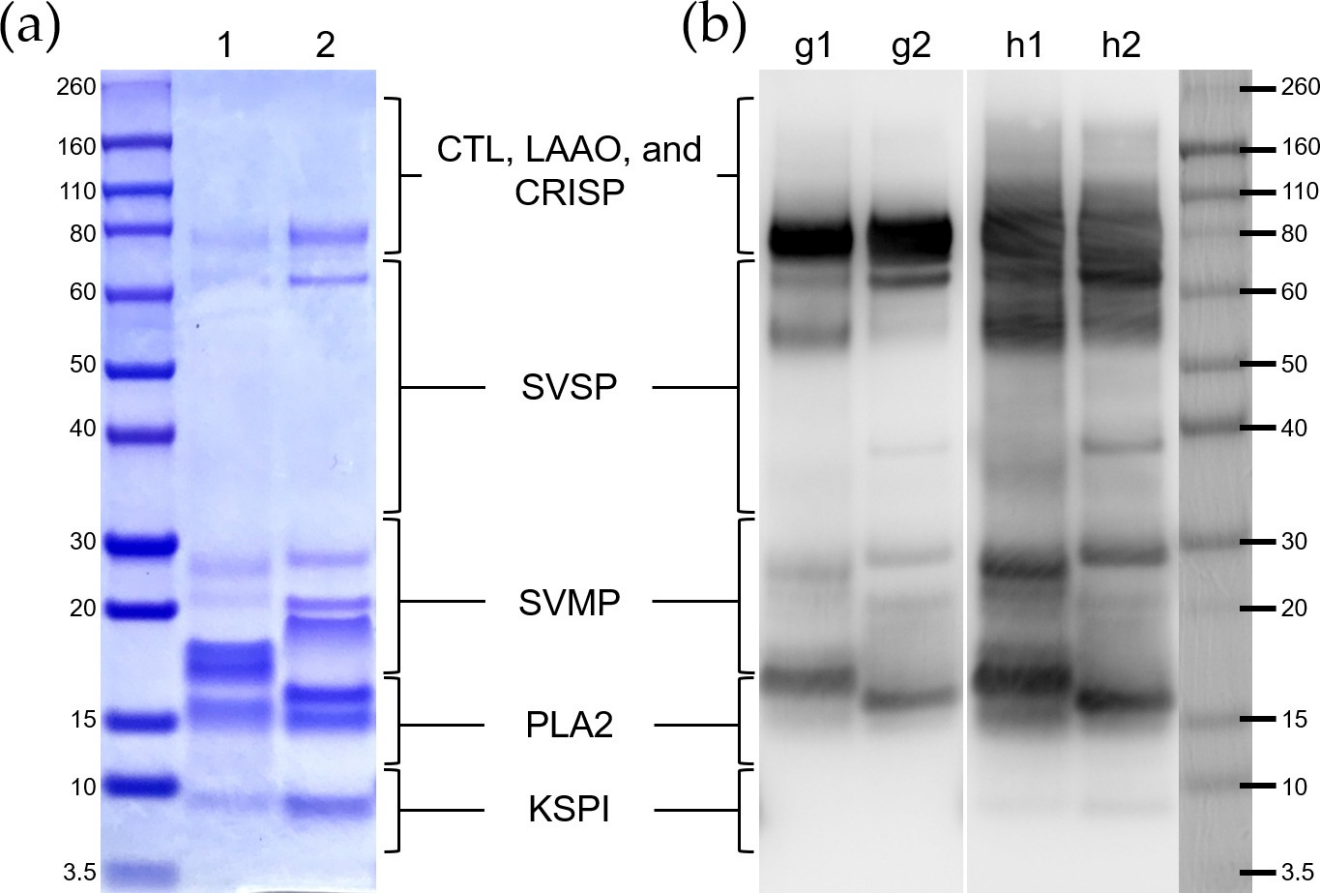
The *D*. *russelii* (RVW, lane 1) and *D*. *siamensis* (RVE, lane 2) venom proteins were (a) stained by Coomassie Brilliant Blue and (b) blotted with goose anti-RVW IgY (lane g1, g2) and horse anti-RVE F(ab’)_2_ (lane h1, h2) after electrophoresis. The protein ladder was roughly divided into five clusters and is labelled according to the major toxin families. Acronyms representing the protein families: CTL, C-type lectin-like protein; LAAO, L-amino-acid oxidase; CRISP, cysteine-rich secretory protein; SVSP, snake venom serine protease; SVMP, snake venom metalloproteinase; PLA_2_, phospholipase A_2_; KSPI, Kunitz-type serine protease inhibitor.

Recently, researchers have shown an increased interest in investigating the difference between RVs venoms from various geographical areas based on advanced proteomic techniques [40–42]. We comprehensively referred to these proteomic findings and roughly divided the protein fractions into five clusters according to the relative positions of the corresponding bands after nonreduced electrophoresis, as indicated in Fig 5. The electrophoretic patterns of the proteins in both RVs venoms shared a similar band distribution, but there were differences in the gel band density and a marked difference in the proteins with molecular weights of 15–20 kDa, which were the phospholipase A_2_ (PLA_2_) and snake venom metalloproteinase proteins (SVMP) and represented the most abundant components of RVs venom. (Fig 5A).

To study the venom protein recognition ability of the goose and horse polyclonal antibodies that were dispensed on the ICT-Viper test strip, the protein-antibody interactions were assessed by western blotting. Our data showed that the two kinds of antibodies could recognize most of the venom proteins of RVE and RVW, with some variations. The purified horse anti-RVE F(ab’)_2_ had broader and stronger performance in the detection of snake venom serine protease (SVSP), SVMP, and Kunitz-type serine protease inhibitor (KSPI). The major venom clusters recognized by both the goose anti-RVW IgY and horse anti-RVE F(ab’)_2_ were PLA_2_ and other high molecular weight proteins, such as C-type lectin-like protein (CTL), L-amino-acid oxidase (LAAO), and cysteine-rich secretory protein (CRISP). The two kinds of venoms had similar protein components and induced significant cross-reactivity between the venoms and antibodies raised from geese or horses (Fig 5A & 5B).

### The performance of ICT-Viper in venom detection *in vitro*

**Fig 6:**
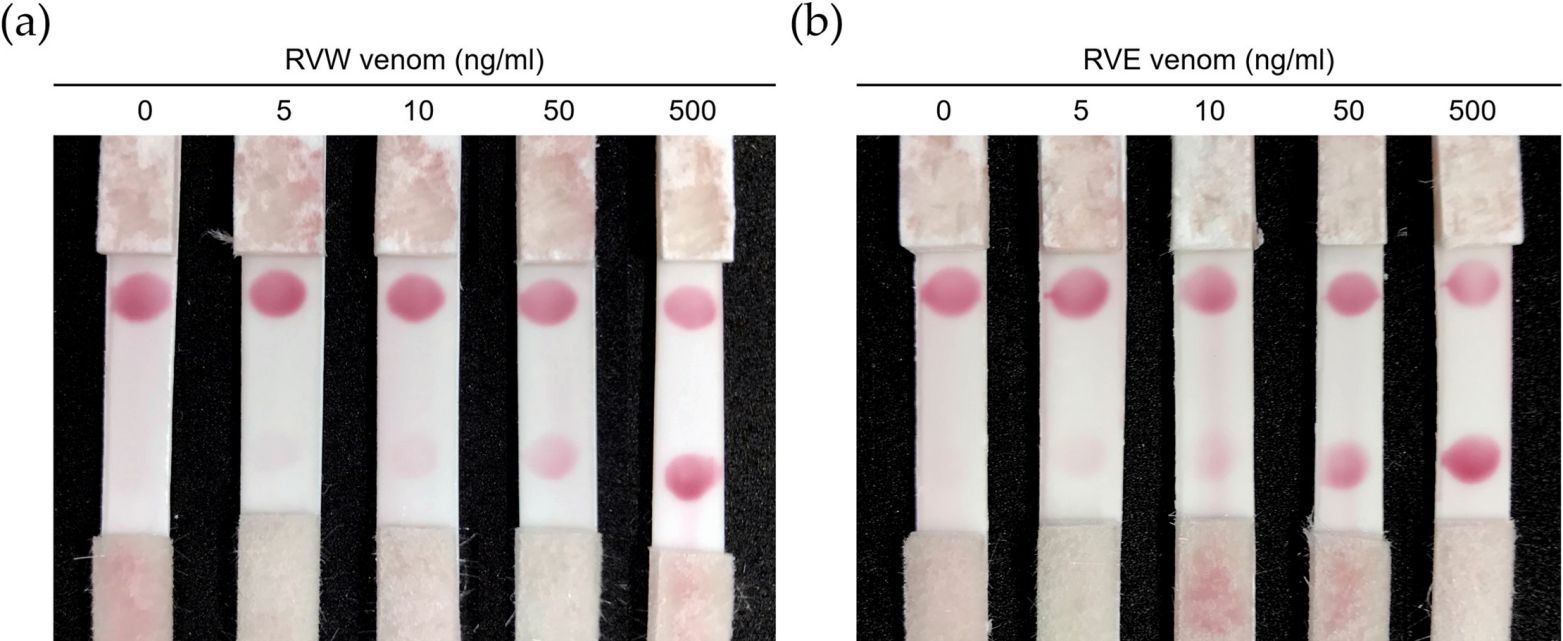
The *in vitro* detection performance of ICT-Viper. The *D*. *russelii* (RVW) (a) or *D*. *siamensis* (RVE) (b) venom samples were dissolved at final concentrations of 0, 5, 10, 50, and 500 ng/ml in fetal bovine serum (FBS) to mimic the viscosity of human serum. The sample volume loaded onto every immunochromatographic strip was equal (90 μl).

**Fig 7:**
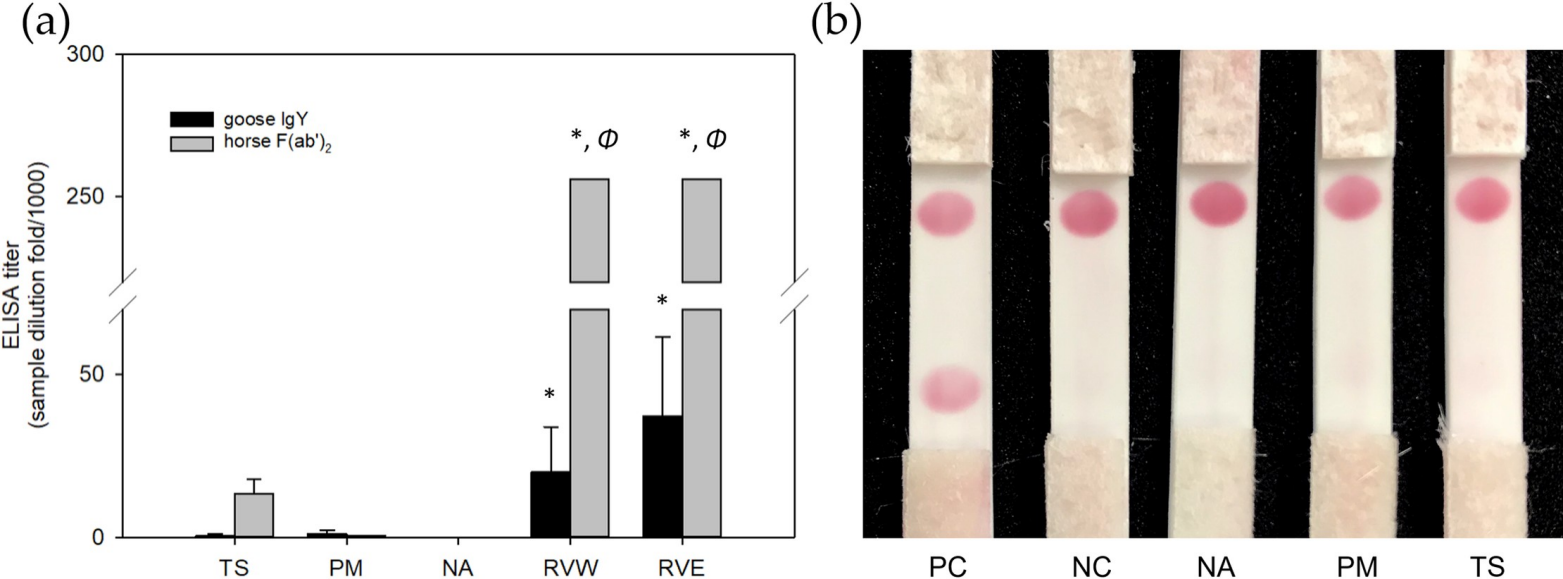
The cross-reaction was illustrated by using indirect ELISA and testing with ICT-Viper. (a) The results of indirect ELISA are presented as the mean ± SD. *, *p* <0.05 versus all heterologous venom samples (TS, PM, and NA). *Φ*, the ELISA titer exceeded the highest sample dilution (1:256,000) used in the analysis. (b) All venom samples were dissolved in fetal bovine serum (FBS) to mimic the viscosity of human serum. The immunochromatographic strips were loaded with a 90 μl sample volume and tested at a concentration of 500 ng/ml. Acronyms: PC, positive control, 100 ng/ml *D*. *russelii* (RVW) snake venom. NC, negative control, FBS only. NA, PM, and TS indicate *Naja atra*, *Protobothrops mucrosquamatus* and *Trimeresurus stejnegeri*.

The ICT-Viper with collocated goose anti-RVW IgY and horse anti-RVE F(ab’)_2_ showed great performance in detecting RV venoms *in vitro*, with the lowest limit of detection of 10 ng/ml (Fig 6). Although there was some cross-reaction between the TS venom and the horse anti-RVE F(ab’)_2_ based on the ELISA assay, the ELISA titers of the goose and horse antivenom in the detection of PM and TS venom, which are derived from the two most common viper snakes leading to envenoming in Taiwan, were far below those resulting from the detection of venom from both RVs (*p* <0.05) (Fig 7A). ICT-Viper also showed accurate detection and negatively reacted with venoms from PM, TS or NA at concentrations up to 500 ng/ml (Fig 7B).

### The performance of ICT-Viper in detection of clinical envenoming

**Fig 8:**
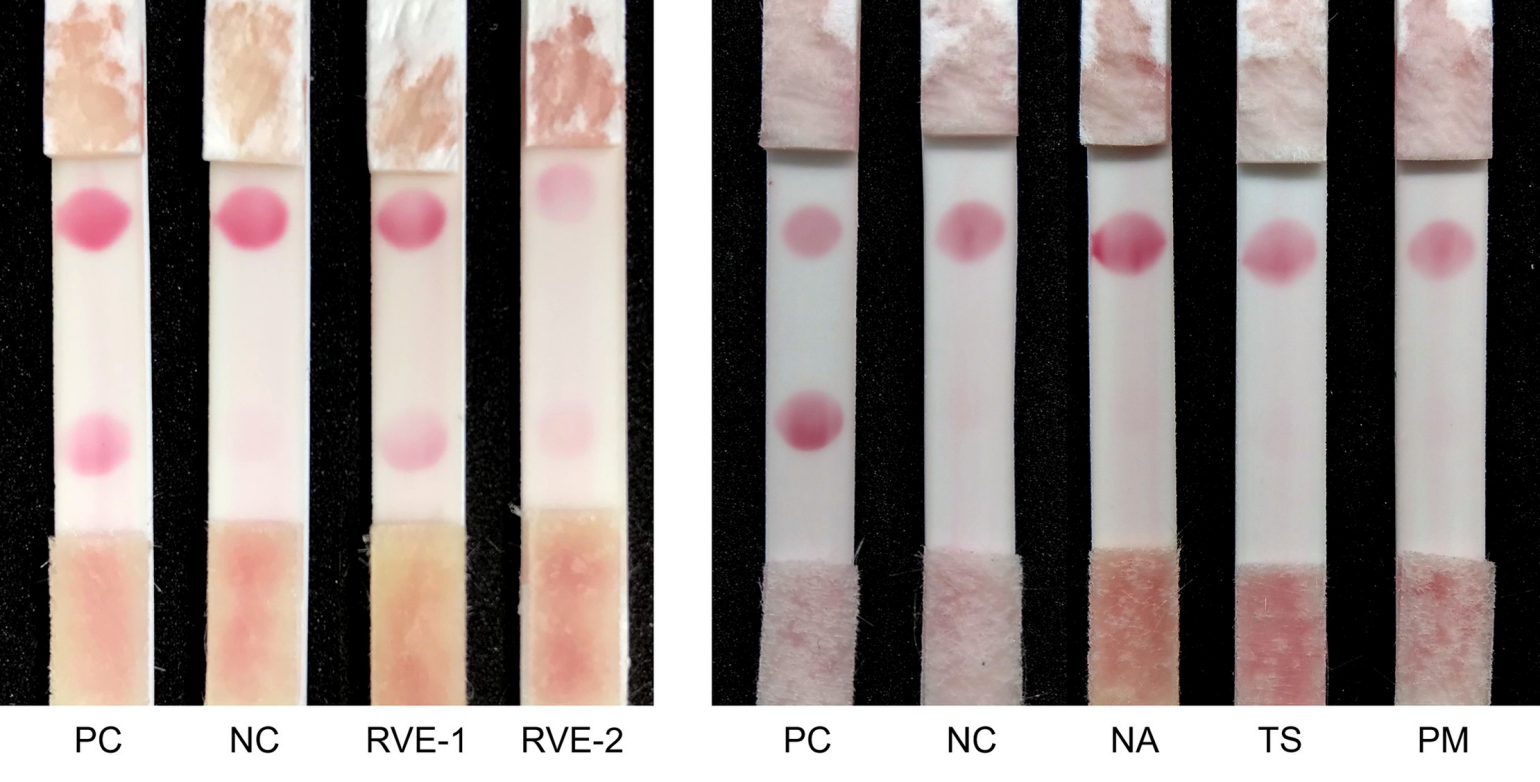
Five stored clinical samples were tested to reveal the performance of ICT-Viper in the differential diagnosis of common snakebites in Taiwan. PC: positive control, addition of 100 ng/ml *D*. *russelii* (RVW) snake venom to healthy human serum donated from volunteers without any snakebite history. NC: negative control, healthy human serum. RVE-1 and RVE-2 indicate serum samples collected from two individual patients, and their detected venom concentration by sandwich ELISA were 13.6 and 3.7 ng/ml respectively. Other snakebite envenomation samples, e.g., NA, TS, and PM, were tested at the same time. The serum concentration of venoms was 459.3, 5.9, and 15.7 ng/ml in NA, TS, and PM sample individually. NA, PM, and TS indicate *Naja atra*, *Protobothrops mucrosquamatus* and *Trimeresurus stejnegeri*.

We also tested the applicability of ICT-Viper in some stored human serum samples from cases of envenoming caused by RVE or other snakes in a previous clinical trial, which had been confirmed by modified sandwich enzyme-linked immunosorbent assay (sandwich ELISA) [8, 20]. The two RVE snakebite samples (RVE-1 and RVE-2) revealed positive results and negative findings for other pit vipers or cobra snake envenoming cases (Fig 8).

### A feasible multi-detection immunochromatography strip (ICT-VC) method to identify Russell's viper and cobra venom

**Fig 9:**
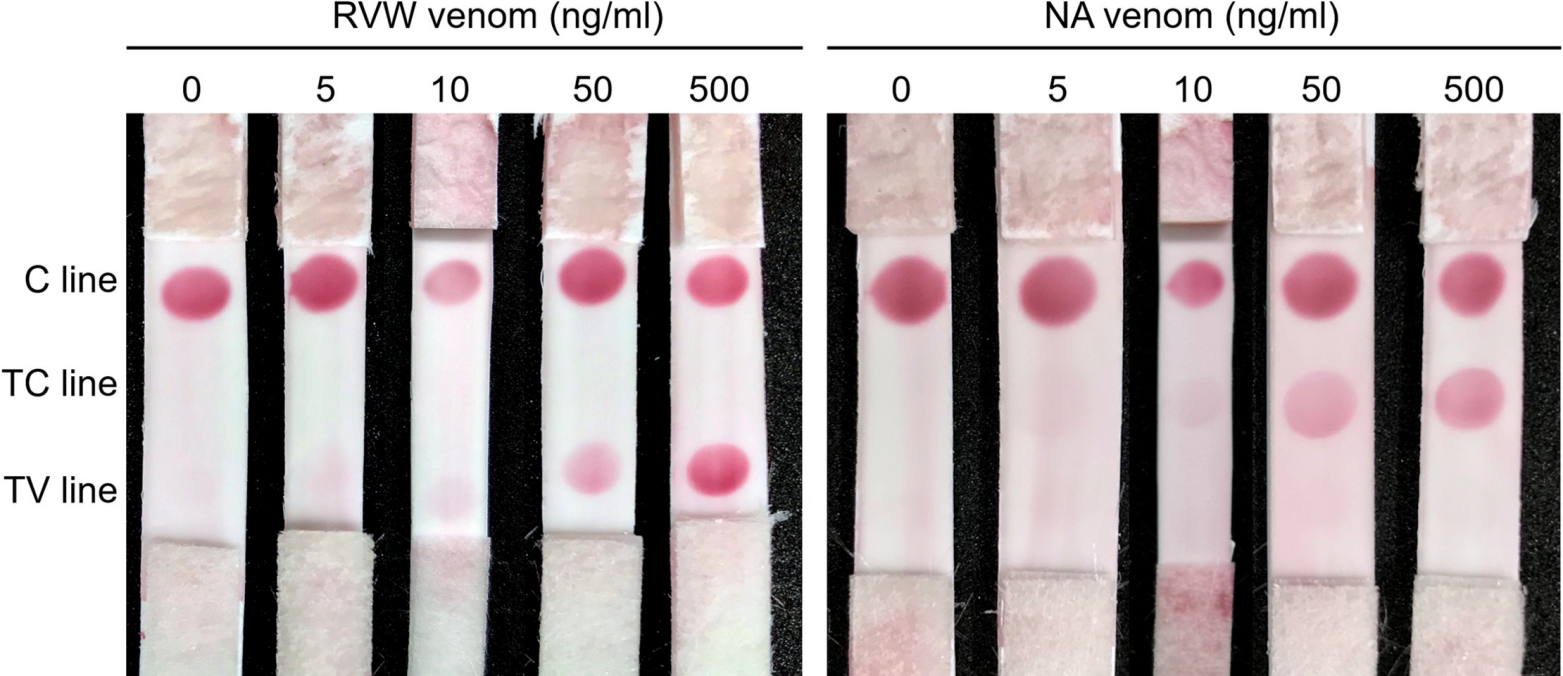
Discernment of *D*. *russelii* (RVW) and cobra venom by using a mixed configuration immunochromatography strip (ICT-VC) with the C line, TC line (cobra test zone), and TV line (RVs test zone). The test venom samples contained 0, 5, 10, 50, and 500 ng/ml venom dissolved in FBS and were loaded according to the conditions described in Fig 6.

Previously, we successfully developed and used an ICT-Cobra kit for the rapid diagnosis of cobra snake bites [21]. Here, we attempted to combine it with ICT-Viper to expand access to testing. The compound strip was successful *in vitro* in detecting the two types of venom individually with a limit of detection of 10 ng/ml (Fig 9).

## Discussion

Avian yolk antibody is a counterpart of mammalian IgG that is present in the egg yolk of birds, reptiles and amphibians and has been identified as a potential accessible, noninvasive and economic source of polyclonal antibodies for therapy and diagnostic tool due to the phylogenetic and systematic distance between avian species and mammals [43]. It has been reported that chickens and ducks immunized with snake venom could produce IgY molecules, which showed great promise as bioanalytical tools in immunoassays [44, 45]. Our experiments proved that goose waterfowl might also be a suitable source for IgY production due to the lack of side effects, easy egg harvest during immunization and simple extraction and purification of high-quality polyclonal antibodies (Figs 3 and 4). Furthermore, the cost of feeding and antibody raising of geese has been much lower than those of horses or sheep [46, 47]. A report indicated that chicken could produce 200 ml egg yolk/kg per month [48], and another report revealed that 5–6 rabbits could produce the comparable amounts of total antibodies as one chicken in a period of 2 weeks [49]. In the light of our experience, about 5 goose eggs (2.77 mg of purified antibodies per egg) could equal to the amounts of purified antibodies from one vial of commercial antivenom (13.57 mg per vial), and 10–15 eggs for 10,000 kits (2.5 μg IgY per kit). During the entire immunizing process, we totally collected 20 high quality goose eggs (Fig 3). This economic benefit is one of the concerns for the development and broad distribution of ICT-Viper in the future.

In general, venom diversification and complexity, which play important roles in antivenom preparation and cancer research, have been noted and suggested to result from ecological environmental pressures, leading to differences in lethality, toxin activity, immunochemical reactivity and antivenom dosage requirements [11, 50, 51]. The characteristic diversity might also impede the correct diagnosis of snakebite envenomation. As shown in Fig 5A, we indeed found a significant difference in the amounts of PLA_2_ and SVMP, the major toxins in RVs venom, between RVW and RVE [39, 41, 42], but similar band formation with different densities for most venom components was notable. Although the relative percentages of the same components in the two species were not exactly the same, the interspecies similarity of RVs venom composition is indeed helpful for the development of pan-RVs diagnostic devices.

It was not surprising that significant cross-reactivity and similar immunogenicity were also found between RVs venom and polyclonal antibodies produced in both horse and goose (Fig 5B). Horse anti-RVE F(ab’)_2_ could recognize more RVW venom proteins than goose anti-RVW IgY, especially PLA_2_ and large molecular weight proteins. In RVs venoms, a kind of low molecular mass toxin family, Kunitz-type serine protease inhibitor (<10 kDa), are poorly immunogenic in antivenom production [52]. Here, these differences in the venom protein recognition of the horse and goose antibodies might be attributed to the large genetic distance between the classes of Aves and Mammalia in the evolutionary tree. In addition, the protocol of immunization, including the dosage and the site of inoculation, and the total times and frequent of injection, might be a reason why the difference caused. Above reasons might also cause the difference in the ELISA titer between the equine F(ab)_2_ and avian IgY in Fig 7A. The similar but slightly different characteristics of the two antibodies might play a decisive role in the performance of ICT-Viper. The precise mechanisms need more studies in the future. We tried to loaded horse anti-RVE F(ab’)_2_ on the conjugated pad to capture more target venom proteins, whereas the goose anti-RVW IgY on the test zone (T line) of ICT-Viper might exclusively recognized the bounded proteins. This configuration finally demonstrated good limit of detection and the absence of false-positive in terms of the cross-reaction (Figs 6 & 7). Moreover, the ICT-Viper could work well to detect venom in cases of RVE envenomation (RVE-1 & 2) while not detecting other Viperidae snakebite envenomations (Fig 8). Although we used the anti-RVW IgY as one component in ICT-Viper production, we did not yet study its meritorious role on differential diagnosis of RVW envenomation from other Viperidae snakebites in India or Sri Lanka. We speculated that less cross-reactivities of proteomics and antivenomics might exist between RVs and pit vipers in Asia. In the study of Tan et al., they had demonstrated some pit viper antivenoms showed negligible immunoreactivity to RVE venom [53]. We anticipate that the ICT-Viper would perform well in detecting and diagnosing RVs snake envenomation in other Asian regions in future investigations.

LFA-based diagnostic devices usually use a monoclonal antibody or purified mammalian antibody to achieve better sensitivity and specificity [54]. Snake venom is an admixture of proteins. It is almost impossible to raise a monoclonal antibody against each toxin, which would increase costs and complicate loading. Although it could detect snakebite envenomation by assaying just one common protein that exists in most venomous snake venoms, such as phospholipase A_2_, it could differentiate nonvenomous from venomous snake bites but not perform species or genus diagnosis [55]. It needs more extensive investigations to raise monoclonal antibodies against genus or species-specific toxins and be used to diagnose snake envenomation on the genus or species level in some regions of the world [56]. For many years, IgY has been studied and recommended for therapeutic or analytical strategies, but it has rarely been collocated with F(ab’)_2_ (an IgG fragment) in LFA diagnostic devices. Our immunochromatography strips were the first attempt to collocate these two antibodies of different origin, which showed good performance by two features. IgY induces less cross-reactivity with human serum protein compared with other mammalian IgG antibodies and leads to the lowest detection interference [35]. Therefore, the collocation of purified goose IgY and equine F(ab’)_2_ resulted in an applicable RVs snake envenomation diagnostic kit.

Although severe coagulopathy and bleeding might be an important clue for the diagnosis of RVs snake envenomation, such envenomation really needs to be differentiated from that caused by other Viperidae snake bites in the Asian region. This is even more complicated and confusing to determine for RVW snake envenomation due to the presence of additional neurotoxic manifestations. To broaden the application of quick test kits, we assembled ICT-Cobra and ICT-Viper together as a multipurpose diagnostic tool, ICT-VC, to address the complex situation in most Asian regions for better snakebite management. The results were good, and the assembled kit accurately detected both venoms individually at concentrations as low as 10 ng/ml *in vitro* (Fig 9).

Sanhajariya et al. have reviewed that the concentration of most snake venoms in serum was above 10 ng/ml soon after being envenomed [57]. We believe that this kit could be applied in most Asian regions where cobra and/or RVs envenomation is the main source of injuries, to differential diagnose snake species quickly and accurately after further investigation.

The results of ICT-Viper application were quite satisfactory from our data. But it still exists some potential challenges. First, the IgY and equine F(ab’)_2_ were polyclonal antibodies, that might exist batch-to-batch variability. It is important to verify the stability of venom recognition in large-scale production. Second, due to the polyclonal characteristics, there is risk of unexpected cross-reactivity with other snake venoms. Third, although eggs are already a compromised choice instead of mammals, it still brings additional works to raise the geese. Fourth, the rising awareness of animal protection might make the immunization of animals for antibody production more difficult. For decades, some investigators have tried to design some rational antibodies with advanced biomedical techniques. The combinative antibody and small recombinant antibody fragments (e.g. scFv and V_H_H) had proved to neutralize some snake toxins effectively [58–61]. The non-animal-derived antibodies would minimize the use of animals [62, 63], and bring the hope for diagnostic strip development. Lastly, we did not verify the ICT-Viper to test other viperids, such as *Echis carinatus*, *Hypnale hypnale* or *Gloydius spp*., which distribute and are epidemiologically important in most of South-East Asia [9]. More regional cooperation and investigation are needed before the kit could be applied in these areas.

Correct snake species identification in cases of snake envenomation is the cornerstone of snakebite management. Our practical immunochromatography strip including ICT-Viper and ICT-VC would contribute to the control of this neglected tropical disease. Avian IgY might be a potential source of antivenom for either diagnostic tools or therapeutics. A large-scale clinical trial to prove its feasibility will be essential in the future. We hope that our promising results can help to meet the global need for a snakebite diagnostic and to achieve this objective before 2030, which was the deadline set by the WHO.
